# Determining Factors of Alarm Fatigue among Nurses in Intensive Care Units—A Polish Pilot Study

**DOI:** 10.3390/jcm12093120

**Published:** 2023-04-25

**Authors:** Katarzyna Lewandowska, Wioletta Mędrzycka-Dąbrowska, Lucyna Tomaszek, Magdalena Wujtewicz

**Affiliations:** 1 Department of Anaesthesiology and Intensive Care Nursing, Medical University of Gdansk, 7 Debinki Street, 80-211 Gdansk, Poland; 2 Department of Specialist Nursing, Faculty of Medicine and Health Sciences, Kraków Academy of Andrzej Frycz Modrzewski, St. Gustawa Herlinga-Grudzińskiego 1, 30-705 Kraków, Poland; 3 Institute of Tuberculosis and Lung Diseases, Rabka-Zdrój Branch, 34-700 Rabka-Zdrój, Poland; 4 Department of Anaesthesiology and Intensive Therapy, Medical University of Gdansk, 17 Smoluchowskiego Street, 80-214 Gdansk, Poland

**Keywords:** alert fatigue health personnel, clinical alarms, critical care nursing, ICU, nurses

## Abstract

Introduction: With the development of medical technology, clinical alarms from various medical devices, which are rapidly increasing, are becoming a new problem in intensive care units. The aim of this study was to evaluate alarm fatigue in Polish nurses employed in Intensive Care Units and identify the factors associated with alarm fatigue. Methods: A cross-sectional study. The study used the nurses’ alarm fatigue questionnaire by Torabizadeh. The study covered 400 Intensive Care Unit nurses. The data were collected from February to June 2021. Results: The overall mean score of alarm fatigue was 25.8 ± 5.8. Participation in training programs related to the use of monitoring devices available in the ward, both regularly (ß = −0.21) and once (ß = −0.17), negatively correlated with nurses’ alarm fatigue. On the other hand, alarm fatigue was positively associated with 12 h shifts [vs. 8 h shifts and 24 h shifts] (ß = 0.11) and employment in Intensive Cardiac Surveillance Units—including Cardiac Surgery [vs. other Intensive Care Units] (ß = 0.10). Conclusion: Monitoring device alarms constitute a significant burden on Polish Intensive Care Unit nurses, in particular those who do not take part in training on the operation of monitoring devices available in their ward. It is necessary to improve Intensive Care Unit personnel’s awareness of the consequences of overburdening and alarm fatigue, as well as to identify fatigue-related factors.

## 1. Introduction

Clinical alarms of monitoring devices in Intensive Care Units (ICUs) are one of the most important methods to warn personnel of direct or potential threats to patients’ health and lives [1]. Monitoring technology in the ICU environment is addressed directly to nurses [2]. The average number of alarms generated by one patient which a nurse responds to during their shift is from 150 to 400 [3]. Responding to alarms constitutes 35% of a nurse’s working time in an ICU [4]. The excessive amount of sounds coming from devices causes sensory overburdening during attempts to verify the significance of a given alarm, which can contribute to fatigue in nurses. The very process of recognizing, assessing and confirming an alarm significantly increases general overburdening at work even if no intervention is necessary [5]. Alarms resulting in a clinical response or intervention constitute only 5–13% of alarms in the monitoring systems [6]. A large number of false alarms is still a problem that is difficult to solve in clinical practice [7].

There are three categories of false alarm: false clinical alarms (e.g., when a patient, while mechanical ventilation is being disconnected, takes a deep spontaneous breath exceeding the breathing volume alarm limit), false technical alarms (e.g., movement artifacts) and false alarms caused by an intervention (e.g., directly by nurses) [8]. The absence of a filter to identify repeating, insignificant and/or false alarms before healthcare professionals are alerted can contribute to the sensory overburdening of medical staff, which is known as alarm fatigue [7]. This is confirmed by the results obtained by Petersen et al. where 88% of nurses agreed that nuisance alarms occur too often and 96% say alarms disrupt patient care [9].

Fatigue caused by alarms is increasingly recognized as a serious problem related to patient safety in contemporary clinical practice [10]. The specificity of work in ICUs led critical care nurses to report low positive patient safety attitudes [11]. Due to the fact that patient safety is a priority in the quality of health care, it is worth raising the awareness of nursing staff on the issue of alarm fatigue [5,12]. This phenomenon is characterized by a delayed response or a lack of response to clinical monitoring alarms due to, among other reasons, nurses’ desensitization to excessive monitoring alarms [13]. In total, 81% of nursing staff declare that their fatigue is due to an excess of false alarms [14]. Factors that contribute to alarm fatigue include work-related and personality-related factors. Work-related factors include, among others, the ICU environment, the length of shift and the number of patients per nurse. In turn, personality-related factors include individual traits of nurses and demographic factors, including, marital status, job seniority and education [15,16]. The ICU environment is a contributing factor to fatigue due to the busy, intense and stressful environment and the variety of alarms that result from the advancement of the therapeutic process [17].

This study is the first project in Poland where alarm fatigue among ICU nurses is examined. The aim of the study was to evaluate alarm fatigue in Polish ICU nurses and identify the factors associated with alarm fatigue.

## 2. Methods

### 2.1. Study Design

It was a cross-sectional study.

### 2.2. Setting and Ethical Considerations

The study was conducted among 400 Polish ICU nurses in the period from February to June 2021. The researchers followed the appropriate legal rules and bioethical principles of the Declaration of Helsinki, giving due regard to the Strengthening the Reporting of Observational Studies in Epidemiology (STROBE) criteria. The protocol of the study was approved by the Bioethics Committee of the Medical University of Gdansk (NKBBN/60/2021).

### 2.3. Participants

The study involved female and male nurses of ICUs from all of the 16 Polish voivodeships. The respondents agreed to take part in the study voluntarily and filled in the questionnaire. The participants did not receive any incentive or payment for their participation in the study. The inclusion criteria was their consent to participate in the study and their status of an ICU nurse. Lack of Polish citizenship was a criterion for exclusion. Participants were informed that by completing the questionnaire they consented to participate in the study. The questionnaire was fully anonymous, and it was impossible to identify the participants for the data they provided.

### 2.4. Variables

The study used a diagnostic survey with a questionnaire technique (Computer-Assisted Web Interview). The researchers used the original version of the nurses’ alarm fatigue questionnaire by Torabizadeh [18]. The questionnaire comprised 13 questions. Answers to each question could be given on the basis of the 5-degree Likert scale: “always”, “usually”, “sometimes”, “rarely”, “never”. Scoring from 0 to 4 was granted to particular items in the questionnaire, except for items 1 and 9, which were scored the other way round. The range of scores in the questionnaire was from 8 (minimum) to 44 (maximum), where the higher score indicated a greater impact of alarm fatigue on nurses’ performance. It is necessary to point out that the researchers obtained the author’s consent to use the tool, as well as her result interpretation guidelines. The author of the questionnaire was contacted via ResearchGate. A translation of the scale from English to Polish (English forward translation) was prepared by two independent translators. The Polish version of the scale was discussed by a group of experts and the original version was supplemented with demographic questions concerning those factors that are likely to predispose a person to alarm fatigue. An empty field was also added for the respondents’ comments. The next step was to perform a “backward” translation from Polish into English and to compare both versions of the scale (backward translation into English). The test of the reliability of the Nurses’ Alarm Fatigue Questionnaire in the original version was the Cronbach’s alpha (which was 0.91) [18]. The Cronbach’s alpha values in this study were also satisfactory (0.72).

The electronic form was sent to ICU head nurses, who were requested to distribute it among their subordinates and on trade websites. Head nurses were found via the websites of medical centers with ICUs. The participants were informed that the questionnaire was addressed solely to ICU nurses and that the moment the questionnaire was completed the respondent was deemed to have agreed to take part in the study. The introduction to the questionnaire described the study and its goals. The questionnaire could be completed in approximately 5 min. Sampling was by convenience and using a snowball sampling method.

### 2.5. Outcomes

The primary outcomes described the alarm fatigue experienced by nurses employed in ICU. The secondary outcomes reported factors related to nurses’ alarm fatigue.

### 2.6. Study Size

Assuming that the population of nurses employed in intensive care units as of November 2022 in Poland was 9174 (data obtained from the National Consultant in Anesthesia and Intensive Care Nursing), a structure rate of 50% and a confidence level of 95%, it was estimated that 369 people would constitute the minimum sample size (estimation error of 5%).

### 2.7. Data Analysis

Statistical analysis was carried out with the Statistica 13.3 package (TIBCO Software Inc. (2017), Kraków, Poland). Statistical significance of intergroup differences in the distributions of categorical variables (presented as number and percentage) was determined with a Chi-square test. The Shapiro–Wilk test was used to verify whether a continuous variable had a normal distribution (*p* > 0.05). Statistical characteristics of continuous variables, when they had a normal distribution (alarm fatigue), were presented as the mean and standard deviation. If data were not normally distributed (age, job seniority, participants’ work experience and the number of beds in the ICU) median and quartiles (Q25; Q75) were reported in addition. The power and direction of the relationship between two variables (the alarm fatigue scores and age; job seniority and the number of beds in the ICU) were determined on the basis of the Spearman correlation coefficient (R). The Student’s *t* test was used to compare the means of alarm fatigue between two groups, whereas ANOVA was used to compare the means among three or more groups. The post-hoc test (Tukey’s for different N) was used to determine the significant pair(s) after the ANOVA was found to be significant. Before applying the post-hoc test, the homogeneity of variances among the groups was tested (Levene’s test > 0.05) [19].

A multivariable linear regression model was calculated to find the relationships between nurses’ alarm fatigue and independent variables such as: gender, age, job seniority, education, type of ICU, the number of beds in the ICU, the structure of the ward, shift length, alarm management systems, and/or participation in training programs related to the use of monitoring devices available in the ward. Independent variables with a *p*-value ≤ 0.1 in simple linear regression models were selected and introduced into the backward step-wise regression (the equal probability value for entry and removal was 0.05). The following assumptions for calculating multiple regression were met: a linear relationship between dependent and independent variables (based on scatterplots), multivariate normality (Shapiro–Wilk test *p* > 0.05), no multicollinearity (Variance Inflation Factor < 1.2), homoscedasticity (based on plotting standardized residuals against predicted values) and independence of observation (the Durbin–Watson statistic = 2) [20]. The results of all multivariable regression models were presented as standardized regression coefficients (ß) and their 95% confidence intervals (CI), and partial R^2^. The threshold of statistical significance for all tests was set at *p* = 0.05.

## 3. Results

### 3.1. Characteristics of Participants

The mean age of the study participants was 35.7 ± 10.0 years (median 33 [27; 45]) and over 50% of them were ≤35 years old (*n* = 223). The majority of participants were women (88.0%), had a Master of Science in Nursing (60.0%), worked 12 h shifts (76.7%) and had a specialization in “Anesthesiology Nursing and Intensive Care” (40.0%). Mean job seniority was 12.5 ± 10.3 (median 10 [4; 21]), while the study participants’ work experience in an ICU was 9.6 ± 8.6 years (median 6 [3; 15]). Over 65% of nurses worked in an ICU ≤ 10 years. Most of the participants were employed in an ICU in the Pomeranian voivodeship (*n* = 186; 46.5%). The mean number of beds in the ICU was 9.7 ± 4.8 (median 10 [6; 12]).

### 3.2. Nurses’ Alarm Fatigue Questionnaire Responses

Responses to individual questionnaire questions are presented in Table 1 and Supplement S1. The analysis of “always/usually” responses suggests that noise in ICUs is a serious burden to nurses. The majority of the personnel pay attention to noise (72.4%; *n* = 290) and are sure that some noise comes from alarms generated by monitoring devices (75.5%; *n* = 302). Alarm noises are annoying to around 40% of nurses (*n* = 153), in particular if nurses feel bad: “When I’m upset and nervous, I’m more responsive to alarm sounds” (59.2%; *n* = 237). As a result of repetitive alarms, some nurses become indifferent to alarms (22.7%; *n* = 91) or impatient (39.5%; *n* = 158). Every third nurse is not able to focus on their professional duties due to alarm sounds (34%; *n* = 136). Almost every tenth nurse (9.5%; *n* = 38) turns alarms down at the beginning of each shift. Responding to alarms is disturbed in the case of a large work overload (37.7%; *n* = 151) or visitors coming to patients (7.7%; *n* = 31).

No relationship was identified between clinical best practices and the hospital having (or not) an alarm management system in place (*p* > 0.05). Alarm management best practices are “always/usually” followed by over 60% of the respondents, which means that they regularly adjust alarm thresholds based on patients’ clinical symptoms (χ^2^ = 2.53; *p* = 0.64) or make their response to respirator alarms dependent on alarm levels (low—yellow, high—red) (χ^2^ = 5.15; *p* = 0.27).

### 3.3. Overall Alarm Fatigue Scores

**Figure 1:**
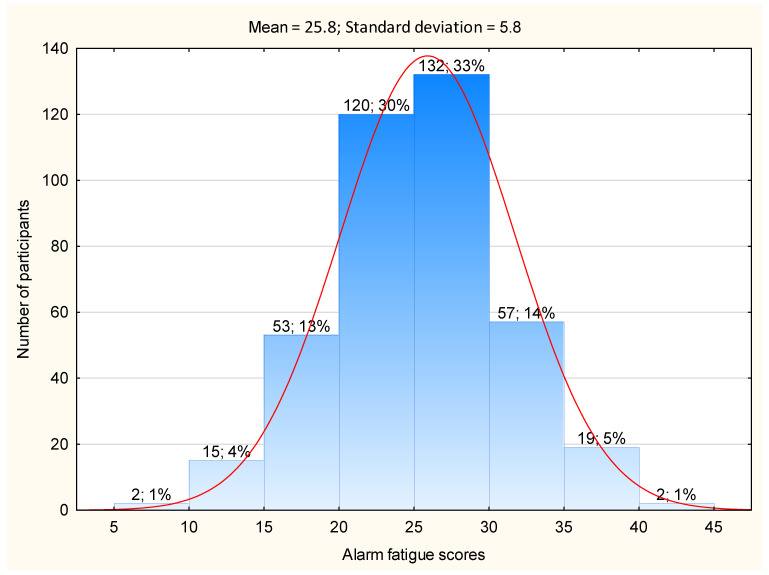
Histogram of the distribution of overall nurses’ alarm fatigue scores.

The distribution of alarm fatigue scores is shown in Figure 1. The overall mean score of alarm fatigue was 25.8 ± 5.8 (minimum 9/44 and maximum 41/44). There was a significant difference (*p* < 0.05) in the mean alarm fatigue scores between nurses who participated in training programs related to the use of monitoring devices available in the ward (regular, only once) and those who did not participate in such programs. It is worth noting that as many as 45.7% (*n* = 183) of the respondents declared that they had never participated in a course in this field (Table 2). The results of this study revealed that there were no significant correlations between either the alarm fatigue scores and the age of the participants (R = −0.06; t = −1.24; *p* = 0.21) or their job seniority (R = −0.04; t = −0.81; *p* = 0.41), or between alarm fatigue and the mean number of beds in the ICU (R = −0.01; t = −0.16; *p* = 0.87).

### 3.4. Factors Related to Nurses’ Alarm Fatigue

Table 3 shows the multiple linear regression model for nurses’ alarm fatigue. The model is statistically significant (*p* = 0.000004) and explains no more than 6% of the variance in the nurses’ alarm fatigue. The contribution to this model comes from four variables. Participation in training programs related to the use of monitoring devices available in the ward, both regularly and once, negatively correlated with nurses’ alarm fatigue. On the other hand, 12 h shifts (vs. 8 h shifts and 24 h shifts) and employment in Intensive Cardiac Surveillance Units—including Cardiac Surgery (vs. other ICUs)—were positively associated with alarm fatigue.

## 4. Discussion

This study found that the sounds of monitoring devices in ICU wards are the main cause of noise, which is a factor in causing nervousness among nursing staff. Alarm fatigue depends on participation in training programs related to the use of monitoring devices available in the ward, shift length, and type of ward.

Work with patients in the ICU causes an overload associated with high responsibility and care demands [21]. In the years 2012–2014 the ECTI Institute’s reports listed the alarm hazards in the context of patients’ safety as the first of the top ten technology hazards in health care [22]. Today, ECRI reports that the technology-induced hazard is the Alarm, Alert, Notification Overload of medical staff [23]. Our study confirms that device alarms have a negative impact on staff well-being, causing impatience or even indifference and unresponsiveness. The fact that they are activated repeatedly makes it difficult for as many as one in three nurses to focus on their professional duties, and it is likely that one in ten nurses silences the alarms at the beginning of their shift for this very reason. Silenced alarms can cause a patient’s declining condition to go unnoticed. A short-term overlooking of a period of clinical instability in a patient with sufficient physiological reserve may have no health consequences. However, prolonged ignoring of vital sign decompensation may lead to the exhaustion of compensatory mechanisms and have dire consequences for the health and lives of patients [24]. The consequences of an adverse event resulting from alarm fatigue fall on both the patient and the nursing staff. Staff involved in serious adverse events become “second victims” by living with guilt, experiencing post-traumatic stress and/or leaving the profession [25]. Therefore, the hazards of excessive alarms in the ICU environment can pose a risk to the safety of the patient as well as the medical staff.

Alarm fatigue and overload significantly reduce the well-being of ICU nurses. This study proved that alarm fatigue among ICU nurses was 25.8 ± 5.8. A lower fatigue tendency was exhibited by nurses from Iran in the study conducted by Asadi et al., 2022, at the same time when the Polish study was carried out, by the use of the same questionnaire (19.08 ± 6.26), but on a smaller sample of nurses (*n* = 140) [26]. Greater fatigue among the respondent nurses was described by US researchers, who examined the levels before and after alarm management training. Alarm fatigue results increased from the pre-implementation level (30.59 ± 5.56) to the post-implementation level (32.60 ± 4.84), which indicates that there was no significant difference between those periods [27]. A survey of 77 ICU nurses using self-reported questions by researchers in Korea showed alarm fatigue at 24.3 ± 4.0 out of 35. The staff paid particular attention to “constantly disturbed by alarms” and “frequent false alarms that lead to lowered attention or response to alarms” [28].

The results of this study indicate that training in managing the alarms of monitoring devices is an important factor in reducing alarm fatigue in nurses. Asadi et al. confirm this by reporting that the fatigue in nurses who were trained in working with ventilators and alarm settings was significantly lower [26]. According to the authors of the 2022 review, education may be key to minimising fatigue [10]. A 2019 project conducted in China revealed similar findings. Alarm fatigue among ICU staff was shown to decrease following alarm management training based on action planning theory [2]. A similar view is presented by a 2019 study assessing nursing staff fatigue before and after an educational programme. All nurses (*n* = 40) who participated in the study agreed that they felt anxious due to clinical alarms, and after the educational intervention the percentage decreased to 50% [1]. However, the absence of a correlation between training and staff fatigue was observed by Seifert. The researchers succeeded in reducing the number of alarms, but the nurses’ fatigue did not decrease [27]. Conscious alarm management by nurses involves reducing false alarms, and decreasing and preventing alarm fatigue [29]. There is no standardised model for training in the use of monitoring devices and alarm management in Poland. The findings described leave no doubt that there is a need for continuously raising the awareness of nursing staff on the consequences of alarm fatigue [30] and prevention methods. This can be achieved through training, which should be targeted primarily at nurses starting work in the ICU. Zhao et al. note that nurses working in the ICU for no more than 10 years had less knowledge of alarm fatigue than those with more seniority [31]. There are also reports that show that raising awareness of Alarm Fatigue should start as early as during nursing education [32].

In addition to alarm management training, the length of a shift and the type of ICU are important factors affecting alarm fatigue. The results of the study suggest greater alarm fatigue in nurses employed in the 12 h system and in the Cardiac ICU. A study published in 2021 by Storm and Chen confirms a predisposition to alarm fatigue among nurses working 12 h shifts [33]. Tirvienė et al. note that Lithuanian nurses working 12 h day shifts exhibited greater mental fatigue than those working 24 h shifts. On the other hand, 24 h shifts posed a risk of greater physical fatigue [34]. The noise of false alarms is considered the most stressful sound in the ICU, and can contribute to fatigue and irritability [35,36]. Noise in the hospital environment should not exceed 40 db during both day and night. Noise levels in the ICU are higher than the recommendations, with monitor alarms at 67 dB and ventilators at 69 dB [37,38]. In turn, prolonged exposure to loud and frequent alarms can lead to a subconscious disregard of sounds [39]. In a qualitative study, Simson et al. quote an ICU nurse “You can sit next to a ringing bell and not hear it, because it’s overwhelming” [40]. Based on this study, we can conclude that during a 12 h shift the staff will be more affected by the noise, which will lead to fatigue, and during a 24 h shift they may ignore the sounds. There are no studies in the literature confirming higher alarm fatigue among Cardiac ICU nurses. Since fatigue is associated with the number of alarms, the number of alarms per day in the Cardiac ICU was 128 [41]. By comparison, there are 142 alarms per patient per day in General ICUs and 96 in Neonatal ICUs [42]. What is also interesting is that the specific patient group for the Cardiac ICU—patients on LVADs (left ventricular assist devices)—generated 549 arrhythmia alarms over 593 h of monitoring, 98% of which were false [43]. An increasing number of clinical alarms may result in increasing fatigue [9]. The above study only verified an area of factors that were not related to personal predispositions of staff. In 2021, an observational study was published in which alarm fatigue was investigated using a subjective workload assessment technique and the impact of boredom, apathy and lack of confidence was measured using questionnaires. Factors contributing to greater fatigue included nurse-to-patient ratio, length of shifts and the priority of tasks. People with personality traits such as openness, conscientiousness and neuroticism also showed greater fatigue [15]. A different finding is presented by a study conducted on a group of ICU staff (doctors and nurses) in 16 hospitals in Lebanon. Identified determinants of alarm fatigue were increased feelings of stress, anxiety, low quality of life and the medical profession [16]. In the future, when designing a study, it is worth considering a precise evaluation of factors and collaboration with psychologists to identify personality traits predisposing to experiencing greater alarm fatigue.

The above pilot study demonstrates the need for further research into the Polish environment on the phenomenon of Alarm Fatigue to improve the quality of work among nursing staff. Alarm fatigue causes work overload in quantitative, qualitative, cognitive and physical areas, which has an overall impact on the well-being of the staff member [44]. There is now a growing body of studies reporting the need to support and nurture a culture of staff well-being, which ultimately leads to an improvement in the quality of patient care and a reduction in medical errors [45]

## 5. Conclusions

The alarms of monitoring devices constitute a significant burden on nurses working in ICU wards. Nurses working 12 h shifts and in Intensive Cardiac Surveillance Units were more tired of alarms. Training seems to reduce the risk of fatigue. Further studies are necessary to confirm the above findings and identify other potential alarm fatigue determinants. In future, it is also worth expanding the questionnaire with other standardized tools that would allow for a more precise assessment of the issue.

### 5.1. Strengths and Limitations

The authors of this study are the forerunners of research on alarm fatigue in Poland, where there are no standardized alarm management programs, and the issue of alarm fatigue is not monitored in any way. This project is the first of its kind to focus on ICU nursing staff in Poland. The hope is that this study will launch a debate on the issue of alarm fatigue and put a spotlight on the threat to patient health in the face of the potential desensitization of nursing personnel. It is also the basis for extending this pilot study to reinforce the strength of the evidence supporting nurses’ well-being in ICU.

This research has limitations. The first one is low diversity among the respondents, a majority of which were women (88%) and residents of the Pomeranian voivodeship (46.5%). In order to carry out a more extensive analysis in the future, the scope of the research should be expanded, and the questionnaires should be distributed in person across various regions in Poland.

Another limitation is the identified small number of factors affecting the Fatigue Alarm. The authors of this study plan to expand the questionnaire to include additional standardized tools allowing for improved identification of factors. This is a pilot study that shows us further paths for the development of the Alarm Fatigue topic. In particular, noticing the small amount of research on alarm fatigue among nursing staff.

### 5.2. Implications for Clinical Practice

ICU nurses are tired from noise generated by monitoring equipment. Therefore, it is necessary to raise their awareness of the potential risks of serious undesired incidents resulting from sensory overburdening. To minimize fatigue, it is necessary to encourage nurse leaders to implement regular training on alarm management in clinical practice. It is worth considering cooperation between engineers and nurses to reduce false alarms. Attention should be paid also to the fact that almost half of the nurses (45.7%) were not trained in the operation and management of alarms in monitoring devices. Hospital administrators should pay more attention to the role of nurses in the medical monitoring system. One good practice is to improve awareness among nurse leaders to enable them to implement training programs for a greater number of ICUs in the future, in order to improve alarm management capacities and reduce fatigue in nurses [2]. Cooperation between nurses and engineers to optimize alarm algorithms for particular patients should also be considered. The clinical adjustment of bed alarm settings will contribute significantly to the reduction of false alarms [25]. The excessive number of false alarms reduces nurses’ vigilance and trust of monitoring equipment, which in turn poses a risk to patients [1].

## Figures and Tables

**Table 1:** A nurses’ alarm fatigue questionnaire—response results.

No.	Statement	Always	Usually	Sometimes	Rarely	Never
1	I regularly readjust the limits of alarms based on the clinical symptoms of patients	90 (22.5)	160 (40.0)	87 (21.7)	54 (13.5)	9 (2.2)
2	I turn off the alarms at the beginning of every shift	8 (2.0)	30 (7.5)	49 (12.2)	82 (20.5)	231 (57.7)
3	Generally, I hear a certain amount of noise in the ward	151 (37.7)	139 (34.7)	68 (17.0)	37 (9.2)	5 (1.2)
4	I believe much of the noise in the ward is from the alarms of the monitoring equipment	88 (22.0)	214 (53.5)	76 (19)	22 (5.5)	0 (0.0)
5	I pay more attention to the alarms in certain shifts	100 (25.0)	149 (37.2)	113 (28.2)	26 (6.5)	12 (3.0)
6	In some shifts the heavy workload in the ward prevents my quick response to alarms	41 (10.2)	110 (27.5)	160 (40.0)	69 (17.2)	20 (5.0)
7	When alarms go off repeatedly, I become indifferent to them	11 (2.7)	80 (20.0)	123 (30.7)	114 (28.5)	72 (18.0)
8	Alarm sounds make me nervous	61 (15.2)	92 (23.0)	163 (40.7)	68 (17.0)	16 (4.0)
9	I react differently to the low-volume (yellow) and high-volume (red) alarms of the ventilator	124 (31.0)	140 (35.0)	73 (18.2)	43 (10.7)	20 (5.0)
10	When I’m upset and nervous, I’m more responsive to alarm sounds	105 (26.2)	132 (33.0)	95 (23.7)	51 (12.7)	17 (4.2)
11	When alarms go off repeatedly and continuously, I lose my patience	42 (10.5)	116 (29.0)	142 (35.5)	68 (17.0)	32 (8.0)
12	Alarm sounds prevent me from focusing on my professional duties	34 (8.5)	102 (25.5)	142 (35.5)	89 (22.2)	33 (8.2)
13	At visiting hours, I pay less attention to the alarms of the equipment	7 (1.7)	24 (6.0)	59 (14.7)	127 (31.7)	183 (45.7)

Results presented as absolute numbers (percentages).

**Table 2:** Overall alarm fatigue scores according to participants characteristics.

Variable	Frequency (Percent)	Mean (Standard Deviation)	Test Statistic, *p* Value
Gender			
• Female	352 (88.0)	26.0 ± 5.8	t = 1.67 *p* = 0.09
• Male	48 (12.0)	24.5 ± 5.4	
Education			
• Registered Nurse	28 (7.0)	24.6 ± 5.7	F = 0.65 *p =* 0.52
• Bachelor in Nursing	132 (33.0)	26.0 ± 5.9	
• Master of Science in Nursing	240 (60.0)	25.8 ± 5.7	
Specialization in “Anesthesiology nursing and intensive care”			
• Yes	176 (44.0)	25.6 ± 6.1	t = 0.47 *p =* 0.64
• No	224 (56.0)	25.9 ± 5.6	
Type of ward			
• Department of Anaesthesiology and Intensive Care (adults)	252 (63.0)	25.4 ± 6.0	F = 1.46 *p =* 0.21
• Intensive Cardiac Surveillance Unit (including Cardiac Surgery)	64 (16.0)	27.1 ± 5.1	
• Post-Operative Intensive Care Unit	32 (8.0)	26.2 ± 4.3	
• Neurological Intensive Care Unit (including Neurosurgery)	25 (6.2)	26.9 ± 6.5	
• Department of Anesthesiology and Pediatric Intensive Care	27 (6.7)	24.8 ± 5.9	
The structure of ward			
• Open	137 (34.2)	27.3 ± 6.2	F = 2.26 *p =* 0.10
• Close	104 (26.0)	25.9 ± 5.9	
• Mixed	159 (39.7)	26.3 ± 5.4	
Shift length			
• 8 h shifts	19 (4.7)	25.3 ± 6.5	F = 2.42 *p* = 0.09
• 12 h shifts	307 (76.7)	26.1 ± 5.6	
• 24 h shifts	74 (18.5)	24.5 ± 6.4	
The ward has alarm management systems for monitoring devices			
• Yes	317 (79.2)	25.6 ± 5.7	t = −1.54 *p =* 0.12
• No	83 (20.8)	26.7 ± 6.2	
Participation in training programs related to use of monitoring devices available in the ward			
• Regularly	60 (15.0)	23.8 ± 5.3	F = 10.93 *p* = 0.00002 Post-hoc test: Regularly vs. No *p* = 0.0007 Once vs. No *p* = 0.0002
• No	183 (45.7)	27.2 ± 5.9	
• Once	157 (39.2)	24.9 ± 5.4	

**Table 3:** Multiple linear regression analysis for variables predicting for nurses’ alarm fatigue.

Factors	Simple Regression ß (95% CI)	Multiple Regression ß (95% Cl)	Partial R^2^
Participation in training programs related to use of monitoring devices available in the ward—regularly ^reference category: No^	−0.14 (−0.24 to −0.04) *	−0.21 (−0.31 to −0.11) **	0.04
Participation in training programs related to use of monitoring devices available in the ward—once ^reference category: No^	−0.12 (−0.22 to −0.02) *	−0.17 (−0.27 to −0.07) **	0.03
Intensive Cardiac Surveillance Unit—including Cardiac Surgery ^reference category: other ICU^	0.10 (0.005 to 0.20)	0.10 (0.009 to 0.20) *	0.01
12-h shifts ^reference category: 8 and 24-h shifts^	0.11 (0.01 to 0.20) *	0.11 (0.02 to 0.21) *	0.01
24-h shifts ^reference category: 8 and 12-h shifts^	−0.10 (−0.20 to −0.007)	Model: R2 = 0.06; F (4,395) = 7.82; *p* = 0.000004	
The structure of ward—open ^reference category: closed and mixed^	0.10 (−0.006 to 0.20)		
Department of Anaesthesiology and Intensive Care (adults) ^reference category: other ICU^	−0.09 (−0.19 to 0.005)		
Female gender ^reference category: male^	0.10 (−0.01 to 0.19)		

ß—standardized regression coefficient; Cl—confidence interval; R2—adjusted coefficient of determination; *p* < 0.05 *; *p* < 0.001 **; The assumptions for calculating multiple regression were met: Shapiro–Wilk test: *p* = 0.83, The Durbin–Watson statistic = 2; the Variance Inflation Factor < 1.2; ICU—Intensive Care Unit.

## Data Availability

The data that support the findings of this study are available from the main author (K.L.) upon reasonable request.

## Supplementary Materials

The following supporting information can be downloaded at: https://www.mdpi.com/article/10.3390/jcm12093120/s1, Supplemental S1: A nurses’ alarm fatigue questionnaire—response results.
