# Supplementary material for: Determining Factors of Alarm Fatigue among Nurses in Intensive Care Units—A Polish Pilot Study
Source: J Clin Med. 2023 Apr 25;12(9):3120. doi: 10.3390/jcm12093120 (PMC10179395; doi:10.3390/jcm12093120)
Supplement: Supplementary file 1 [file jcm-12-03120-s001.zip › jcm-2361807-supplementary.pdf]

Supplement S1. A nurses' alarm fatigue questionnaire – response results

**No.**	**Statement**	**Mean SD**	**Median Q_{25}; Q_{75}**
**1**	I regularly readjust the limits of alarms based on the clinical symptoms of patients	1.3 ± 1.0	1 [1; 2]
**2**	I turn off the alarms at the beginning of every shift	0.7 ± 1.0	0 [0; 1]
**3**	Generally, I hear a certain amount of noise in the ward	3.0 ± 1.0	3 [2; 4]
**4**	I believe much of the noise in the ward is from the alarms of the monitoring equipment	2.9 ± 0.8	3 [3; 3]
**5**	I pay more attention to the alarms in certain shifts	2.7 ± 1.0	3 {2; 3.5]
**6**	In some shifts the heavy workload in the ward prevents my quick response to alarms	2.2 ± 1.0	2 [2; 3]
**7**	When alarms go off repeatedly, I become indifferent to them	1.6 ± 1.1	2 [1; 2]
**8**	Alarm sounds make me nervous	2.3 ± 1.0	2 [2; 3]
**9**	I react differently to the low-volume (yellow) and high-volume (red) alarms of the ventilator	1.2 ± 1.1	1 [0; 2]
**10**	When I'm upset and nervous, I'm more responsive to alarm sounds	2.6 ± 1.1	3 [2; 4]
**11**	When alarms go off repeatedly and continuously, I lose my patience	2.2 ± 1.1	2 [1.5; 3]
**12**	Alarm sounds prevent me from focusing on my professional duties	2.0 ± 1.1	2 [1; 3]
**13**	At visiting hours, I pay less attention to the alarms of the equipment	0.9 ± 1.0	1 [0; 1]

Results presented as mean (standard deviation; SD) and median and quartiles (Q25; Q75)
